# MicroRNA-200a suppresses the Wnt/β-catenin signaling pathway by interacting with β-catenin

**DOI:** 10.3892/ijo.2011.1322

**Published:** 2011-12-30

**Authors:** JUAN SU, ANLING ZHANG, ZHENDONG SHI, FEIFEI MA, PEIYU PU, TAO WANG, JIE ZHANG, CHUNSHENG KANG, QINGYU ZHANG

**Affiliations:** 1 Department of Gastroenterology, Tianjin Medical University General Hospital, Tianjin 300052; 2 Department of Neurosurgery, Tianjin Medical University General Hospital and Laboratory of Neuro-Oncology, Tianjin Neurological Institute, Tianjin 300052; 3 Tianjin Medical College, Tianjin 300011, P.R. China

**Keywords:** Wnt/β-catenin pathway, microRNA, epithelial mesen-chymal transition, proliferation, invasion

## Abstract

The Wnt/β-catenin signaling pathway is crucial for human organ development and is involved in tumor progression of many cancers. Accumulating evidence suggests that the expression of β-catenin is, in part, regulated by specific microRNAs (miRNAs). The purpose of this study was to determine the expression of a recently identified epithelial to mesenchymal transition (EMT)-associated tumor suppressor microRNA (miR)-200a, in cancer cells. We also aimed to identify specific miR-200a target genes and to investigate the antitumor effects of miR-200a on the Wnt/β-catenin signaling pathway. We employed TOP/FOP flash luciferase assays to identify the effect of miR-200a on the Wnt/β-catenin pathway and we confirmed our observations using fluorescence microscopy. To determine target genes of miR-200a, a 3′ untranslated region (3′ UTR) luciferase assay was performed. Cell viability, invasion and wound healing assays were carried out for functional analysis after miRNA transfection. We further investigated the role of miR-200a in EMT by Western blot analysis. We found fluctuation in the expression of miR-200a that was accompanied by changes in the expression of members of the Wnt/β-catenin signaling pathway. We also determined that miR-200a can directly interact with the 3′ UTR of CTNNB1 (the gene that encodes β-catenin) to suppress Wnt/β-catenin signaling. MiR-200a could also influence the biological activities of SGC790 and U251 cells. Our results demonstrate that miR-200a is a new tumor suppressor that can regulate the activity of the Wnt/β-catenin signaling pathway via two mechanisms. MiR-200a is a candidate target for tumor treatment via its regulation of the Wnt/β-catenin signaling pathway.

## Introduction

Constitutive activation of the Wnt/β-catenin signal pathway promotes uncontrolled cell growth and survival, and can consequently drive cancer formation (1,2). Dysregulated Wnt/β-catenin signaling is a common feature of many malignant tumors of epithelial tissue origin (3–5). In epithelial tumors, mutations in components of the β-catenin destruction complex (such as APC, AXIN and GSK3β) or in the β-catenin gene were shown to contribute to the cytosolic accumulation of β-catenin and the activation of the Wnt/β-catenin pathway (6,7). MicroRNAs (miRNAs) are single-stranded non-coding RNAs of 21 to 23 nucleotides that repress translation or induce cleavage of target mRNAs that are partially complementary to the 3′ or 5′ untranslated regions (UTRs). MiRNAs have recently been implicated in the regulation of tumorigenesis, differentiation, proliferation and survival through the regulation of major cellular pathways (8), especially in epithelial tumors. The relationship between microRNAs and the Wnt/β-catenin pathway in epithelial tumors has become a central point of interest.

Our previous review on the Wnt/β-catenin pathway described multiple genes involved in its regulation, with special focus on the function of miRNAs. Several miRNAs have been found to be regulators, either as oncogenes or tumor suppressor genes that regulate the activity of the Wnt/β-catenin pathway (9). MiR-200a was reported to down-regulate β-catenin-mediated transcription; however, little is known about the mechanism involved in this activity. Here, we investigated whether up- or down-regulation of miR-200a expression was accompanied by changes in the activity of the Wnt/β-catenin signal pathway in gastric adenocarcinoma SGC7901 cells and glioblastoma U251 cells. We show that miR-200a can influence the biological characteristics of SGC7901 and U251 cells by regulating the down-stream targets of Wnt/β-catenin signaling. Furthermore, we confirmed that CTNNB1 is a direct target of miR-200a. We determined that miR-200a is an inhibitor of EMT in SGC7901 cells.

## Materials and methods

### Cell culture and transfection

Human stomach adenocarcinoma cell lines, SGC7901 and U251, were obtained from the Laboratory of Neuro-Oncology, Tianjin Neurological Institute. The cells were cultured in DMEM supplemented with 10% fetal bovine serum (FBS). All cultures were maintained at 37°C in a humidified atmosphere containing 5% CO_2_. The miRNA mimic, miRNA inhibitor and negative control were synthesized by GenePharma (Shanghai, China). For transfection, trypsinized cells were plated in 6-well plates at 2-3×10^5^ cells per well. MiRNA transfections were performed using Lipofectamine 2000 (Invitrogen, Carlsbad, CA, USA). For each well, miRNA (100 pmol) in 250 μl of serum and antibiotic-free medium was mixed with 5 μl of Lipofectamine 2000 in 250 μl of the same medium and allowed to stand at room temperature for 20 min. The mixture was then added to cells and after 4 h the medium was changed to complete medium.

The mimic and inhibitor sequences were: miR-200a mimic sense: 5′-UAA CAC UGU CUG GUA ACG AUG U-3′; anti-sense: 5′-AUC GUU ACC AGA CAG UGU UAU U-3′; negative control sense: 5′-UUC UCC GAA CGU GUC ACG UTT-3′; anti-sense: 5′-ACG UGA CAC GUU CGG AGA ATT-3′; miR-200a inhibitor: 5′-ACA UCG UUA CCA GAC AGU GUU A-3′; inhibitor negative control: 5′-CAG UAC UUU UGU GUA GUA CAA-3′.

### Real-time PCR analysis

Total RNA was extracted using TRIzol Reagent (Invitrogen) according to the standard protocol. A nanodrop spectrophotometer (Gene) was used to detect the concentration of total RNA. Total RNA (1 μg) was used to synthesize cDNA by reverse transcription using MMLV reverse transcriptase (Promega Corp., Madison, WI, USA), according to the manufacturer’s instructions. Real-time PCR analysis was performed to determine the abundance of miR-200a in SGC7901 and U251 cells 48 h after transfection with miR-200a mimic or inhibitor or scrambled negative control. The expression of u6 was used as an internal control. We performed qRT-PCR for 40 cycles, comprising 95°C for 10 min, 95°C for 15 sec, 65°C for 30 sec, 72°C for 30 sec and an extension at 72°C for 10 min.

Real-time PCR analysis was also performed to determine β-catenin mRNA levels and data were normalized to GAPDH, which was used as an internal control. Both reverse transcription and qRT-PCR primers were purchased from GenePharma.

### Plasmid construction

TOPflash and FOPflash reporters contain wild-type (WT) and mutated TCF-4 consensus binding sites, respectively, and are widely used to evaluate β-catenin-dependent signaling events that drive the expression of TCF. These reporters have been described previously (10). The wild-type 3′ untranslated region (UTR) of the CTNNB1 gene, containing predicted miR-200a target sites, and a mutated CTNNB1 3′ UTR in which the miR-200a target sites were mutated were inserted into the *Xba*I and *Fse*I sites of the pGL3 control vector (GenScript, Nanjing, China) and were named pGL3-CTNNB1 and pGL3-CTNNB1-mt, respectively.

### Luciferase assays

Cells (0.5-1×10^5^ cells/well) were plated in 24-well plates 1 day prior to transfection. The miR-200a mimic/inhibitor transfection was performed according to the Lipofectamine 2000 instructions (Invitrogen), and 48 h after reporter plasmid transfection, luciferase activity was measured using a luciferase reporter assay system (Promega).

### Western blot analysis

After transfection, cells were washed with ice-cold phosphate-buffered saline (PBS) three times and were lysed for 30 min on ice in RIPA buffer in the presence of a proteinase inhibitor cocktail, then centrifuged at 12,000 rpm for 15 min at 4°C. Proteins were harvested and 40 μg from each sample was subjected to SDS-PAGE separation, and then transferred to a PVDF membrane (Millipore, USA). The membrane was incubated with primary antibodies against β-catenin, ZEB1, ZEB2 (Abcam; 1:1000 dilution), E-cadherin, N-cadherin, Tcf-4, Fra-1, MMP-9 and Cyclin D1 (Santa Cruz; 1:1000 dilution), followed by incubation with an HRP-conjugated secondary antibody (Zhongshan Bio, Beijing, China). Specific proteins were detected using a SuperSignal protein detection kit (Pierce, USA). The membrane was stripped and re-probed with a primary antibody against GAPDH (Santa Cruz; 1:1000 dilution).

### Fluorescence microscopy

Twenty-four hours after transfection, cells were plated on glass cover slips and 48 h post transfection the cover slips were washed extensively in phosphate buffered saline (PBS) and fixed with 4% paraformaldehyde in PBS. After additional washing, the cells were permeabilized with 1% Triton X-100 in PBS for 10 min. The cover slips were then washed and blocked with 1% BSA for 30 min. Cells were incubated in the appropriate primary antibodies (β-catenin or TCF-4) overnight at 4°C. Samples were then washed and incubated with species-specific secondary rhodamine-labeled antibodies (TRITC) in PBS (1:100 dilution) for 60 min. Nuclei were stained with DAPI at RT for 10 min and cover slips mounted with Antifade solution prior to imaging on a confocal microscope (Leica microsystems, Heidelberg, Germany).

### Wound healing assay

Cell culture and transfection conditions were optimized to ensure a homogeneous and viable cell monolayer prior to wounding. One day before transfection, equal numbers of SGC7901 cells (2×10^5^) or U251 cells (1×10^5^) were seeded in 6-well plates. When cell confluence reached about 90%, approximately 24 h post-transfection, an artificial homogenous wound was made onto the monolayer using a sterile plastic 200 μl micropipette tip. After wounding, debris was removed by washing cells with PBS. At different time points, cells that migrated into the wounded area or cells with extended protrusions from the wound border were photographed at ×200 magnification under a light microscope.

### Transwell cell migration assay

The top chamber of a transwell chamber was incubated with 60 μl Matrigel diluted with DMEM (1:2, Matrigel: DMEM) at 37°C for 30 min. The Matrigel solidified and acted as the extracellular membrane (ECM) for tumor cell invasion analysis. Transfected cells were trypsinized, adjusted to 5×10^5^/ml in DMEM, and 100 μl of the resuspended cell solution was added to the top chamber above the Matrigel. The bottom chamber was filled with 600 μl of chemoattractant solution. The transwell plate was assembled and incubated at 37°C, in a 5% CO_2_ incubator. After 24 h, the top chamber was removed, and the Matrigel and unmigrated cells were gently scraped with a wet cotton swab. Cells were stained by crystal violet for 3 min, and washed with PBS to remove excess stain. Finally, cells were counted under a light microscope. The average number of migrated cells per field was quantified under high power (x200).

### Flow cytometry

Forty-eight hours after transfection, cells were trypsinized and collected by centrifugation, washed in PBS and fixed with 75% ethanol overnight at 4°C. Cells were then washed twice with PBS, and incubated with 200 μl RNase A (1 mg/ml) at 37°C for 30 min. Cells were then stained in the dark with 800 μl propidium iodide staining solution for 30 min at 4°C. Analysis was performed on a FACSCalibur flow cyto-meter (Bio-Rad, USA).

### MTT assay

Cells were seeded in a 96-well plate at a density of 3000 cells per well, and 24 h before transfection, cells were incubated with 20 μl MTT solution (5 mg/ml). At 24, 48, 72, 96, 120, 144 and 168 h following transfection at 37°C for 4 h, the solution was aspirated, and 200 μl DMSO was added to each well. The Optical density (OD) was measured at a wavelength of 570 nm. The data are presented as the mean ± SD, which are derived from triplicate samples of at least three independent experiments.

### Statistical analysis

A commercially available software package, SPSS16.0, was used for statistical analysis. One-way analysis of variance (ANOVA) and the χ^2^ test was used to analyze the significance between groups. The LSD method of multiple comparisons with parental and control vector groups was used when ANOVA showed statistical significance. Statistical significance was determined at the level of p<0.05.

## Results

### Modulation of miR-200a expression by a mimic and an inhibitor

**Figure 1:**
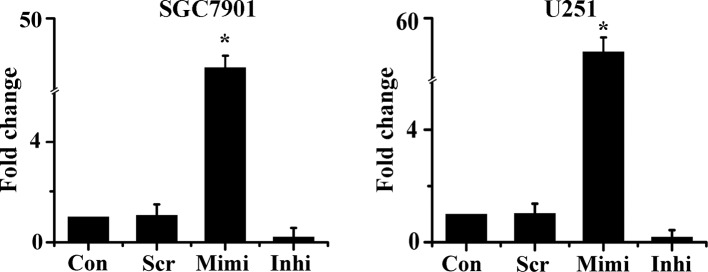
miR-200a expression in SGC7901 and U251 cells. MiR-200a mimic, inhibitor or scrambled control were delivered into SGC7901 and U251 cells. Cells transfected with the miR-200a mimic showed a higher level of miR-200a expression, while cells transfected with miR-200a inhibitor were silenced for miR-200a expression.

To monitor the expression of miR-200a in target cells, the miR-200a mimic, inhibitor and scrambled control were delivered into SGC7901 and U251 cells. The level of miR-200a expression was then examined 48 h after transfection by qRT-PCR. Expression of miR-200a was up-regulated by approximately 40-fold in cells transfected with the miR-200a mimic. Meanwhile, in cells transfected with the miR-200a inhibitor, miR-200a expression was reduced by about 80% compared with control (Fig. 1). These results were used as the basis of the subsequent experiments.

### Relationship between miR-200a expression and activity of the β-catenin/Wnt signaling pathway

**Figure 2:**
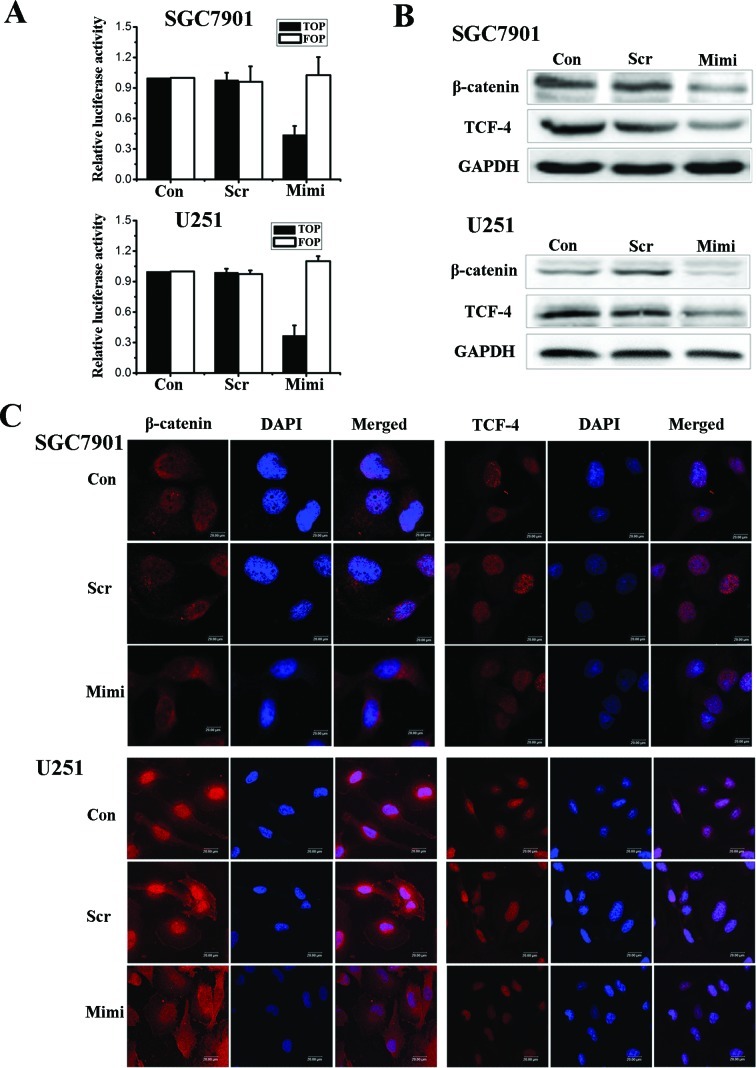
Effect of miR-200a on the activity the Wnt/β-catenin signaling pathway. (A) The luciferase reporter assay using TopFlash and FopFlash vectors was utilized to study β-catenin TCF/LEF promoter activity. The TopFlash reporter vector has 6-TCF binding sites, while FopFlash has mutated TCF binding sites. SGC7901 and U251 cells were co-transfected with different expression vectors as indicated. MiR-200a mimic treatment decreased β-catenin TCF/LEF promoter activity. (B) Up-regulation of miR-200a decreased β-catenin and TCF-4 expression. (C) Immunofluorescence assay for β-catenin indicates that the location of β-catenin in cells shifts from nuclear to cytoplasmic when the expression of miR-200a was increased, and that TCF-4 expression decreased in the nucleus compared to control vector.

Recent studies have reported that miR-200a targets the mRNA of the E-cadherin repressor proteins, ZEB1 and ZEB2. This results in an increase in the level of E-cadherin available for binding to β-catenin and induces formation of the cell-cell adhesion complex (11). We considered that miR-200a may play an important role in regulating the activity of the Wnt/β-catenin pathway. In an effort to determine the relationship between the expression of miR-200a and the activity of the β-catenin/Wnt pathway, we first employed TOPflash and FOPflash reporters, which are widely used to evaluate β-catenin-dependent signaling activity, to evaluate the effects of miR-200a on Wnt/β-catenin signaling in SGC7901 and U251 cells. The luciferase activity of the cells changed as we hypothesized (Fig. 2A). Wnt/β-catenin signaling was inhibited when the level of miR-200a was up-regulated. We then used Western blot assays to investigate the expression levels of β-catenin and TCF-4 proteins (Fig. 2B). Fluorescence microscopy of β-catenin and TCF-4 showed that the location of β-catenin in cells shifts from nuclear to cytoplasmic when the expression of miR-200a increased. At the same time, TCF-4 levels decreased in the nucleus (Fig. 2C).

**Figure 3:**
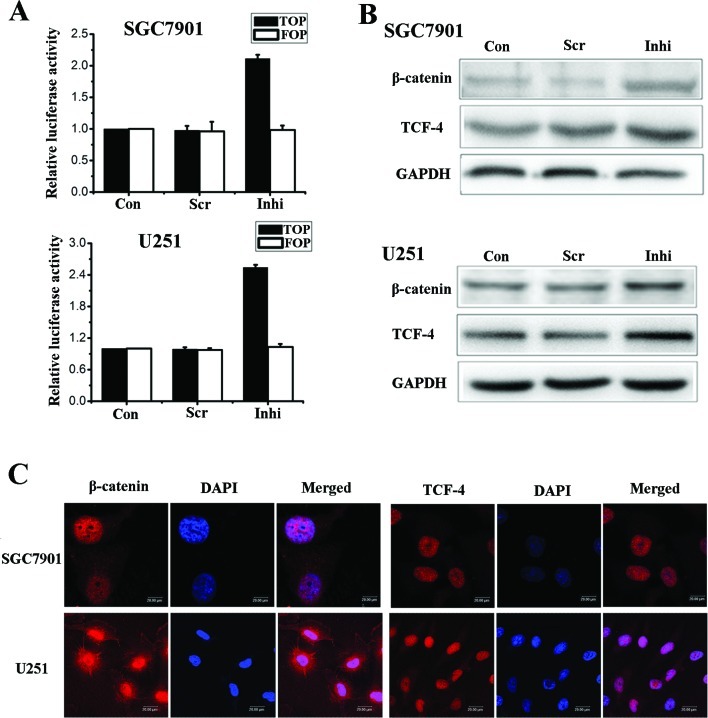
Modulation of the Wnt/β-catenin signaling pathway by miR-200a inhibitor in SGC7901 and U251 cells. (A) Knockdown of miR-200a expression resulted in the induction of TopFlash luciferase activity, indicating the transcriptional activity of the β-catenin/TCF complex. Knockdown of miR-200a expression had no effect on FopFlash luciferase activity. (B) Western blot analysis of β-catenin and TCF-4 in cells treated with miR-200a inhibitor. (C) Lower levels of miR-200a cause nuclear accumulation of β-catenin in both SGC7901 and U251 cells, while levels of TCF-4 increased in the nucleus compared to control vector.

We also used the miR-200a inhibitor to down-regulate the expression of miR-200a in SGC7901 and U251 cells. Using luciferase assays, Western blot analysis and fluorescence microscopy we showed that Wnt/β-catenin signaling activity was negatively correlated with the level of miR-200a (Fig. 3).

**Figure 4:**
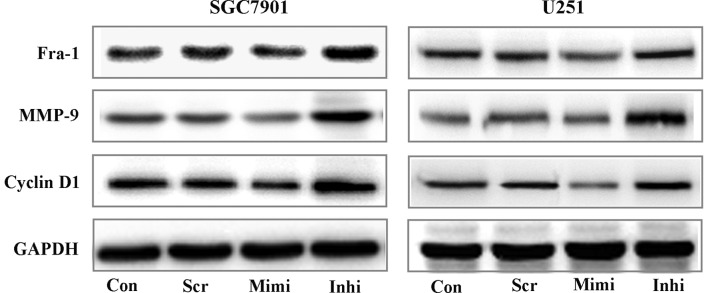
Western blot analyses of proteins in the Wnt/β-catenin signaling pathway and of downstream Wnt/β-catenin signaling pathway targets, such as Fra-1 and Cyclin D1.

It is well known that the Wnt/β-catenin pathway has essential functions in the regulation of cell growth and differentiation. Here, we used Western blot assays to investigate the expression of some downstream targets of Wnt/β-catenin signaling, such as Fra-1, Cyclin D1 and MMPs (12–14) (Fig. 4). These results showed an important correlation between miR-200a expression and activity of β-catenin/Wnt signaling. We therefore postulate that miR-200a affects the biological activity of tumor cells.

### Regulation of tumor cell activity by miR-200a

**Figure 5:**
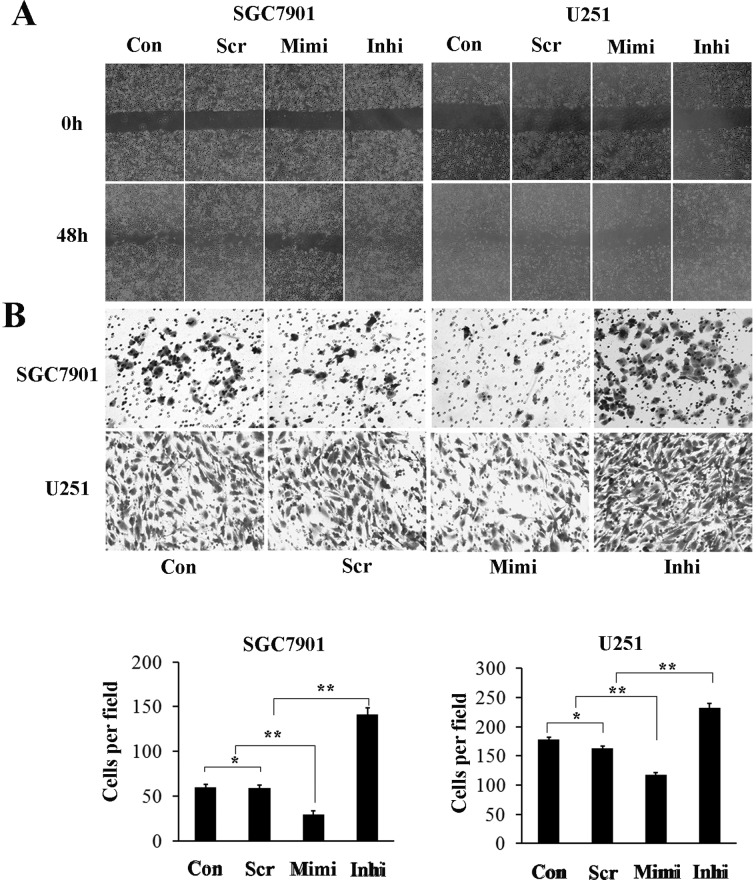
miR-200a affects migration and invasion of the tumor cells. Measurement of cell migration by ‘wound-healing’ assay and Transwell assay. Transient over-expression of miR-200a inhibited the migration of SGC7901 and U251 cells. Data shown are the mean and standard deviation from analyzing three fields per sample in triplicate (^*^p>0.05, ^**^p<0.05).

We next investigated a functional outcome for the miR-200a-mediated suppression of β-catenin/Wnt signaling. The expression level of miR-200a clearly influenced the biological activity of SGC7901 and U251 cells. Therefore, we investigated two major biological activities of tumor cells, namely migration and invasion potential and growth ability. Migration and invasion potential are important biological characteristics of malignant tumor cells. Representative micrographs of wound healing assay and of transwell filters are shown in Fig. 5. The number of cells invading through the matrigel in the miR-200a mimic group was significantly decreased (29.3±4.1), while in the miR-200a inhibitor group it was increased (141.0±7.5) compared to control (59.7±3.0) and scrambled control (58.7±3.8) groups. The invasion activity was inhibited by approximately 40% in the miR-200a mimic group (117.7±8.5) and increased (232.3±13.3) in the miR-200a inhibitor group compared with the control (178.3±11.3) and scrambled control (163.0±14.0) groups (Fig. 4B). These results suggest that high levels of miR-200a inhibit the migration and invasion capacity of SGC7901 and U251 cells, while low levels of miR-200a has the opposite effects.

**Figure 6:**
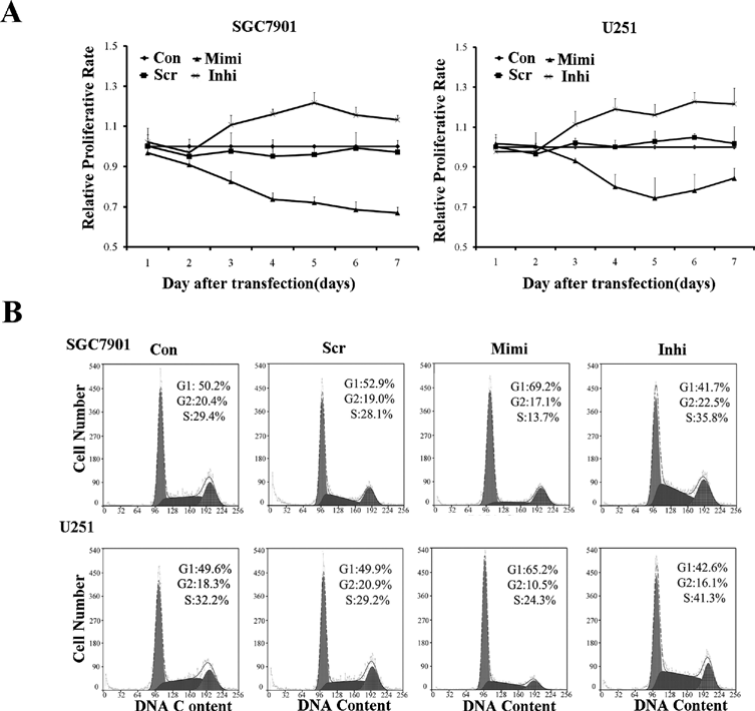
Effect of miR-200a on cell proliferation MTT assay showing that cells transfected with the miR-200a mimic proliferated at a significantly lower rate than controls. The G0/G1 phase fraction in control, scrambled-control treated cells, miR-200a mimic group and miR-200a inhibitor group cells was 50.2%, 52.9%, 69.2% and 41.7% in SGC7901 cells and 49.6%, 49.9%, 65.2% and 42.6% in U251 cells, respectively.

The proliferation rate of variously transfected SGC7901 and U251 cells was measured using the MTT assay. The miR-200a mimic group proliferated at a significantly lower rate than the other groups. Cell cycle analysis confirmed these results. The miR-200a mimic led to G0/G1 entry, while the miR-200a inhibitor blocked G0/G1 entry (Fig. 6).

### Target validation of miR-200a

MicroRNA-200a, a member of the miR-200 family, stands out as an inhibitor of EMT. Direct evidence for the EMT-inhibitory actions of miR-200a has been revealed in several cancer cell lines, including nasopharyngeal carcinoma, endometrial serous adenocarcinomas, bladder cancer and meningiomas (15–18). The up-regulation of miR-200a in NRK52E cells was shown to down-regulate the expression of TGF-β2, via direct interaction with the 3′ UTR of TGF-β2 (19), which can induce EMT and reduce the invasiveness in meningiomas (18). Up-regulation of miR-200a also decreased cellular invasion and metastasis in nasopharyngeal carcinoma cells (15). As described above, miR-200a affects the phenotype of the SGC7901 and U251 cells, although determination of the underlying mechanism requires further investigation.

**Figure 7:**
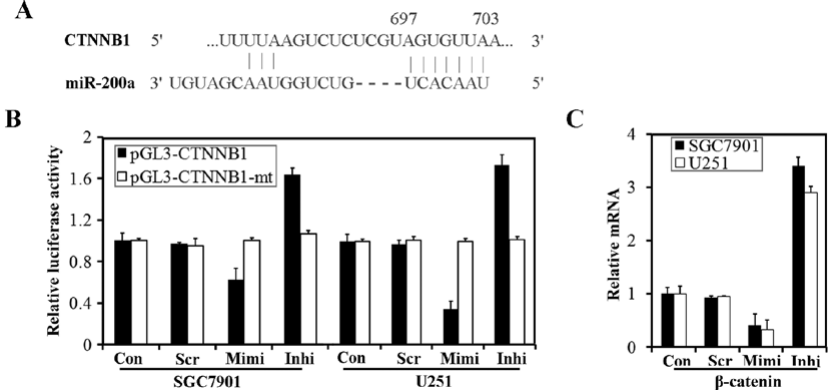
MiR-200a down-regulates CTNNB1 mRNA and protein. (A) The human CTNNB1 3′ UTR and target sites were predicted by TargetScan. (B) Luciferase reporter assays indicated that miR-200a regulates β-catenin expression by targeting the putative target sites. (C) β-catenin mRNA and protein were up-regulated in the presence of miR-200a inhibitors and down-regulated in the presence of the miR-200a mimic in both SGC7901 and U251 cells.

CTNNB1 (the gene which encodes β-catenin) has been suggested to be a target of miR-200a (18,20). According to TargetScanHuman 5.1, the 3′ UTR of CTNNB1 contains predicted seed regions for miR-200a and miR-141 (Fig. 7A). To determine whether endogenous miR-200 could target the 3′ UTR of CTNNB1 in SGC7901 and U251 cells, the 3′ UTR of CTNNB1 and a mutated CTNNB1 3′ UTR were cloned into a modified pGL-3 control vector, placing it downstream of the luciferase coding sequence. We delivered pGL3-CTNNB1 and pGL3-CTNNB1-mt into cells transfected with the miR-200a mimic or inhibitor. Luciferase assays revealed that over-expression of miR-200a could significantly reduce luciferase activity, while down-regulation of miR-200a caused an enhancement of luciferase activity. However, transfection of pGL3-CTNNB1-mt had no effect on luciferase activity of the cells (Fig. 7B). We have identified β-catenin, encoded by CTNNB1, as a target protein for miR-200a. β-catenin mRNA was also down-regulated by transfection of the miR-200a mimic (Fig. 7C).

### Increasing miR-200a levels induces mesenchymal to epithelial transition (MET) in SGC7901 cells

**Figure 8:**
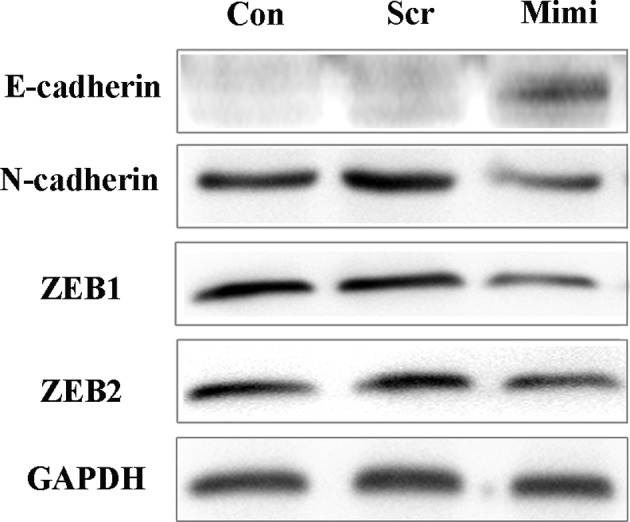
Level of EMT-associated proteins after SGC7901 cells were transfected with miR-200a mimic or inhibitor, determined by Western blotting.

Recently, ZEB1 and ZEB2 have both been suggested to be targets of miR-200a, and miR-200a was demonstrated to cause changes in E-cadherin expression (16,21,22). To determine whether the expression of E-cadherin is also under the control of miR-200a in SGC7901 cells, we used the miR-200a mimic to over-express miR-200a, and Western blot analysis was performed to detect the expression of ZEB and cadherin/catenin complexes (Fig. 8). Over-expression of miR-200a reduced ZEB1, ZEB2 and N-cadherin protein levels, while E-cadherin protein levels were increased, consistent with earlier reports (15,16,21,22).

## Discussion

This study highlights two different mechanisms by which miR-200a regulates the activity of β-catenin. First we showed that miR-200a can inhibit the activity of the Wnt/β-catenin signaling pathway. Then we found that miR-200a can regulate the expression of β-catenin through not only direct interaction with the 3′ UTR of CTNNB1, but also via interaction with the cadherin/catenin complex. In an effort to further investigate the effect of miR-200a on the phenotype of tumor cells, we used epithelial tumor cell lines, SGC7901 and U251, and found that over-expression of miR-200a significantly inhibited SGC7901 and U251 cell growth, invasion and induced G0/G1 phase arrest, while reduced expression of miR-200a promoted tumor cell growth and invasion. The induction of apoptosis by inhibiting the activity of the Wnt/β-catenin signaling pathway in tumor cells was not detected in our studies and further investigations are required to explain the role of miR-200a in tumor cells.

As the most common primary brain tumor, gliomas account for more than 70% of all primary central nervous system neoplasms. Accumulating evidence suggests that aberrant activation of Wnt/β-catenin signaling is involved in glioma development and progression (23). We demonstrated that up-regulation of miR-200a down-regulated the expression of β-catenin and affected the activity of the Wnt/β-catenin signaling pathway. Furthermore, at the protein level, our results indicate that miR-200a also regulates some downstream targets of Wnt/β-catenin signaling, such as Fra-1, Cyclin D1, and MMPs. Yue *et al* (24) showed that knockdown of β-catenin by siRNA in human U251 glioma cells inhibited cell proliferation and invasive ability and induced apoptotic cell death; however, in our studies, apoptosis in U251 cells was undetectable after miR-200a-induced β-catenin down-regulation. It has also been reported that the Wnt/β-catenin signaling pathway regulates the early and late stages of apoptosis in other cancer cells (25,26), so our studies did not conclusively demonstrate the mechanisms of miR-200a action in U251 cells, and additional mechanisms are possible, such as regulation through direct target genes other than β-catenin, or even through other signaling pathways.

We identified for the first time in gastric adenocarcinoma SGC7901 cells that over-expression of miR-200a reduced levels of ZEB1, ZEB2 and N-cadherin and increase E-cadherin levels. ZEB1 and ZEB2 are transcription factors that activate EMT by binding to E-box elements present in the E-cadherin promoter, suppressing transcription. While E-cadherin has a widely acknowledged role in cell-cell adhesion, it also functions as an invasion suppressor protein. During EMT E-cadherin is down-regulated, while N-cadherin is induced and β-catenin is released from junctional complexes and is translocated to the nucleus (27–29). The cadherin-associated protein β-catenin also has the potential to regulate cell motility or invasion. Therefore, via regulating the expression of E-cadherin by targeting ZEB1 and ZEB2, miR-200a can inhibit the invasive potential of tumor cells. In addition, using luciferase reporter assays, we demonstrated that the expression of β-catenin was regulated directly by miR-200a in SGC7901 cells. MiR-200a could also regulate tumor cell invasive ability by directly targeting β-catenin. Although β-catenin was originally identified as an integral component of the cadherin adhesion protein complex, it is also an essential intracellular mediator for the Wnt/β-catenin signaling pathway. So it is reasonable for us to consider that the effect of miR-200a on tumor cell growth might be attributed to β-catenin regulation. We used a new luciferase reporter assay called TCF-responsive reporter, or TOPflash, to investigate β-catenin/TCF-dependent transcriptional activity, and we found a satisfactory correlation between miR-200a expression and Wnt/β-catenin signaling pathway activity. In conclusion, we found that in SGC7901 cells miR-200a regulated both the EMT of the cells and the activity of the Wnt/β-catenin signaling pathway.

**Figure 9:**
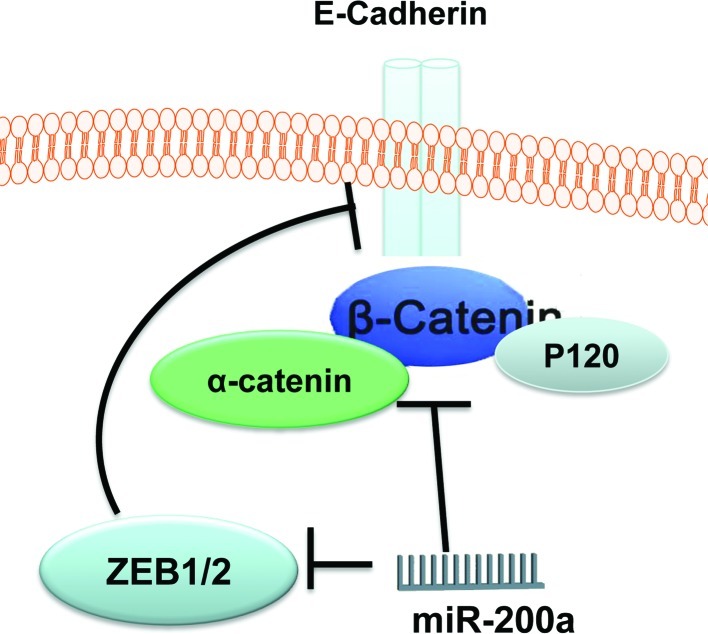
Two mechanisms by which miR-200a regulates the activity of β-catenin. One mechanism is that miR-200a directly interacts with the 3′ untranslated region of β-catenin, another is that miR-200a up-regulates E-cadherin through down-regulation of its target gene, ZEB1/2, which then influences the activity of Cadherin/Catenin complexes.

In summary, β-catenin is an important functional target for miR-200a in gastric adenocarcinoma SGC7901 cells and glioblastoma U251 cells. MiR-200a regulates the activity of β-catenin through two kinds of mechanism (Fig. 9), and up-regulation of miR-200a is a potential therapeutic strategy for glioma and gastric adenocarcinoma.
